# The role of p53 in the alternation of vascular functions

**DOI:** 10.3389/fphar.2022.981152

**Published:** 2022-09-06

**Authors:** Gabriel Hoi-Huen Chan, Enoch Chan, Carsten Tsun-Ka Kwok, George Pak-Heng Leung, Simon Ming-Yuen Lee, Sai-Wang Seto

**Affiliations:** ^1^ Division of Science, Engineering and Health Studies, College of Professional and Continuing Education, The Hong Kong Polytechnic University, Kowloon, Hong Kong SAR, China; ^2^ School of Clinical Medicine, The University of Hong Kong, Pokfulam, Hong Kong SAR, China; ^3^ Department of Applied Biology and Chemical Technology, The Hong Kong Polytechnic University, Kowloon, Hong Kong SAR, China; ^4^ Department of Pharmacology and Pharmacy, The University of Hong Kong, Kowloon, Hong Kong SAR, China; ^5^ State Key Laboratory of Quality Research in Chinese Medicine, Institute of Chinese Medical Sciences, University of Macau, Avenida da Universidade, Taipa, China; ^6^ Research Centre for Chinese Medicine Innovation, The Hong Kong Polytechnic University, Kowloon, Hong Kong SAR, China; ^7^ NICM Health Research Institute, Western Sydney University, Penrith, NSW, Australia

**Keywords:** atherosclerosis, p53, vascular smooth muscle cell, endothelial dysfunction, vascular smooth muscle proliferation, vascular smooth muscle migration

## Abstract

Ageing is a risk factor for many degenerative diseases. Cardiovascular diseases (CVDs) are usually big burdens for elderly, caregivers and the health system. During the aging process, normal functions of vascular cells and tissue progressively lost and eventually develop vascular diseases. Endothelial dysfunction, reduced bioavailability of endothelium-derived nitric oxide are usual phenomena observed in patients with cardiovascular diseases. Myriad of studies have been done to investigate to delay the vascular dysfunction or improve the vascular function to prolong the aging process. Tumor suppressor gene p53, also a transcription factor, act as a gatekeeper to regulate a number of genes to maintain normal cell function including but not limited to cell proliferation, cell apoptosis. p53 also crosstalk with other key transcription factors like hypoxia-inducible factor 1 alpha that contribute to the progression of cardiovascular diseases. Therefore, in recent three decades, p53 has drawn scientists’ attention on its effects in vascular function. Though the role of tumor suppressor gene p53 is still not clear in vascular function, it is found to play regulatory roles and may involve in vascular remodeling, atherosclerosis or pulmonary hypertension. p53 may have a divergent role in endothelial and vascular muscle cells in those conditions. In this review, we describe the different effects of p53 in cardiovascular physiology. Further studies on the effects of endothelial cell-specific p53 deficiency on atherosclerotic plaque formation in common animal models are required before the therapeutic potential can be realized.

## Introduction

With an estimated 17.9 million of people died of cardiovascular diseases (CVDs) in 2019, CVDs are the leading causes of death. The diseases contributed to 32% of the total global deaths. (World Health Organization, 2021). CVDs are group of diseases affecting the cardiovascular system. The cardiovascular system consists of the blood, heart and blood vessels. Blood is in liquid form and constantly being transported inside the body. The heart is a pump that beats about 72 times per minute in human adult and keeps the blood moving throughout the body. The blood vessels are composed of different sizes of arteries, veins and capillaries as conducting duct to transport blood and body fluid. Blood vessels are essential for the transport of molecules such gases, nutrients, wastes, hormones, bioactive substances and also involve in distribution of immune cells and heat in the body. Blood pressure is the determined by the heart pumping and the blood exerted upon the walls of the blood vessels. Normal blood pressure should be less than 120 mmHg for systolic blood pressure and 80 mmg Hg for diastolic blood pressure. Blood pressure higher than 120/80 mmHg is considered to be hypertensive (or a pre-hypertensive state) and it is an indicator for vascular changes. Such vascular changes usually accompanied with altered endothelial cells, vascular smooth muscle cells migration, arterial stiffness, vascular calcification. With such alternations in the arterial walls, blood pressure usually increases (Boron and Boulpaep, 2017; Brown et al., 2018). An elevation in blood pressure lowers cardiac output by increasing afterload, the pressure that opposes the ejection of blood during ventricular systole (Klabunde, 2022). Hypertension is also an important risk factor for various kinds of cardiovascular-associated diseases like heart failure, atrial fibrillation, chronic kidney disease, heart valve diseases, aortic syndromes, and dementia (Fuchs and Whelton, 2020).

**FIGURE 1:**
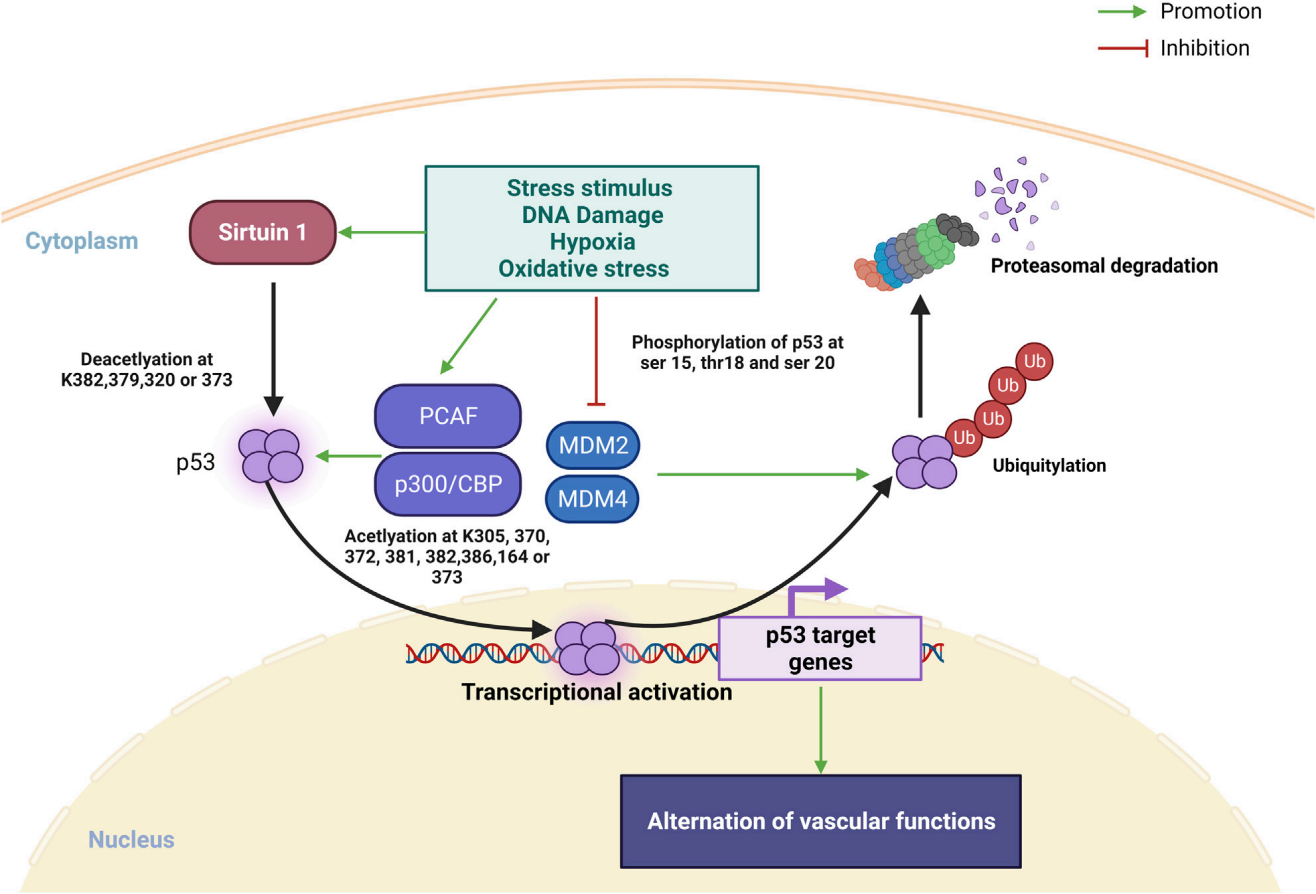
Overview on posttranslational modifications of p53 under stress stimulus. In normal condition, MDM2 and MDM4 promotes p53 ubiquitination leading to p53 degradation in proteasome. Under stress stimulus, sirtuin 1 is activated and deacetylate p53 at K382, 379, 320 or 373 for promoting p53 transcription, PACF and p300/CBP is also activated to promote p53 transcription *via* acetylation at k305, 370, 372, 373, 381, 282, 386 or 164. Ubiquitin proteasome of p53 degradation is inhibited *via* repression MDM2 and MDM4 binding to p53 by stress stimulated phosphorylation of p53 at ser 15, 20, and thr18 (Created with BioRender.com).

Transcription factor p53 is heavily involved in activating or suppressing various genes in response to various stress stimuli, including DNA damage, hypoxia and oxidative stress, and its activation promotes DNA repair, apoptosis, cell cycle arrest, metabolic shifts and autophagy (Vousden and Prives, 2009; Beckerman and Prives, 2010). Moreover, a recent review by Men et al. (2021) also highlighted the regulatory roles of p53 in cardiac function and dysfunction. The regulation of p53 protein is controlled by posttranslational modifications including phosphorylation, acetylation/deacetylation, glycosylation, ubiquitination, SUMOylation and more (Meek and Anderson, 2009) (Figure 1). In unstressed situation, p53 is rapidly turned over by actions of MDM2 and MDM4, which promote poly-ubiquitination, nuclear export and proteasomal degradation of p53. When stimulated by stress, phosphorylation of the amino terminus of p53 prevents the binding of MDM2, leading to stabilization of p53 protein (Meek and Anderson, 2009). Subsequently, lysine residues in the carboxyl terminal the DNA-binding domain of p53 is subject to acetylation by p300/CBP and PCAF, and this acetylation is essential for the stabilization and activation of p53 (Tang et al., 2008; Meek and Anderson, 2009). On the other hand, sirtuin 1, a histone deacetylase, is another inhibitor of p53 activity, causes deacetylation of p53 protein (Vaziri et al., 2001; Kim et al., 2007). SUMOylation also plays a complex role in regulating the turnover and transcriptional activity of p53 (Takabe et al., 2011; Santiago et al., 2013; Dehnavi et al., 2019; Celen and Sahin, 2020).

In this review, we discuss the role of p53 in vascular function, and its potential as a therapeutic target for vascular dysfunction.

## p53 and vascular function

Blood vessels consist of three layers: tunica externa (outermost later), tunica media (middle layer) and tunica intima (innermost later). Tunica externa is mainly made of connective tissues providing support and protection for the vessel. Tunica media comprises mainly of vascular smooth muscle cells and connective tissues. Vascular smooth muscle cells are arranged in helical or circular layers in the intima in larger vessels and as a single circular in small vessels. The contractile state of smooth muscle cells determines the diameter of blood vessels. In healthy blood vessels, when there is change in extravascular environment, local growth factors, vascular active substances, and hemodynamic stimuli initiate structural and functional adaptations of smooth muscles to maintain a normal and stable blood pressure (Renna et al., 2013).

The tunica intima consists of a single-cell layer of endothelial cells. Upon stimulation by acetylcholine, ATP, bradykinin and laminar shear stress, endothelial cells generate physiological amount of nitric oxide (NO) with an enzyme called endothelial nitric oxide synthase (eNOS), and NO stimulates the relaxation of smooth muscle (Furchgott and Vanhoutte, 1989; Fleming and Busse, 2003; Sessa, 2004). The endothelium also produces other vasorelaxant factors including prostaglandin I2 (Toda et al., 2007; Bauer and Sotníková, 2010) and endothelium-derived hyperpolarizing factor (EDHF) (Félétou and Vanhoutte, 1996) as well as vasoconstricting substances such as endothelin (Barton and Yanagisawa, 2019) and thromboxane A2 (Chen, 2018). Endothelial cells also function to keep the inner surface of blood vessel non-adhesive and non-thrombogenic, which is essential to maintain steady blood flow (O'Reilly et al., 2003).

The number of cells within tissue depends on their turnover by apoptosis and cell proliferation, which are highly regulated processes. In normal arteries, the turnover of smooth muscle cells and endothelial cells are low. eNOS inhibits the apoptosis of endothelial cells (Dimmeler et al., 1997; Polte et al., 1997), as well as the proliferation of endothelial cells and vascular smooth muscle cells (Tsihlis et al., 2011). Moreover, laminar shear stress can increase cellular level of p53 within endothelial cells. p53 is stabilized upon phosphorylation by c-Jun N-terminal kinase (JNK). This increase in p53 level leads to cell cycle arrest and the suppression of apoptosis of endothelial cells (Lin et al., 2000). Events such as healing of injuries, skeletal muscle adaptation after exercise or female reproductive cycles trigger angiogenesis (Otrock et al., 2007). Vascular endothelial growth factor (VEGF) promotes angiogenesis through promoting endothelial cell survival, proliferation and migration (Olsson et al., 2006). p53 also play a complex role in regulating VEGF expression. In the presence of an intact p21-Rb pathway, p53 represses VEGF expression. However, in the absence of p21-Rb pathway, which occurs in malignant cells, p53 promotes VEGF expression (Farhang Ghahremani et al., 2013a; Farhang Ghahremani, et al., 2013b).

## p53 and endothelial dysfunction

Endothelial dysfunction is a broad term that refers to the impairment of the normal physiological functioning of the endothelium. It involves, but is not limited to, impaired endothelium-dependent vasodilatation, increased leukocyte recruitment into tunica intima, increased endothelial permeability to lipoproteins, and increased thrombotic property. A key cause of endothelial dysfunction is the reduced bioavailability of eNOS (Thomas et al., 2008).

Apart from reduction of eNOS, DNA damage, mitochondrial dysfunction, oxidative stress, telomere dysfunction and other stressors induce several tumor suppressor genes including p53 and lead to cellular senescence, the permanent state of cell arrest. (Jerafi-Vider et al., 2021; Sun and Feinberg, 2021). Cellular senescence is originally a physiological protective mechanism to prevent the development of tumor, however, when such senescent cells accumulate in number it leads to aging of cells (López-Otín et al., 2013). Senescence of vascular endothelial cells plays an important role in initiation and progression of CVDs (Hohensinner et al., 2016; Jia et al., 2019). Cellular oxidative stress, circulating IGF-1 deficiency, altered calcium signaling, may impair cell functions and contribute to endothelial damage that lead to cerebrovascular diseases and loss of cognitive functions (Tarantini et al., 2017). The senescent cells can remain alive, though accompanied by phenotypical changes, altered metabolism and gene expression. The senescent EC usually are flatter and enlarged with more polypoid nucleus and at the same time with changed structure in cytoskeleton that may lead to angiogenesis, proliferation and migration of the cells (Sun and Feinberg, 2021). Thus, endothelial senescence is an important biomarker for CVDs.

**FIGURE 2:**
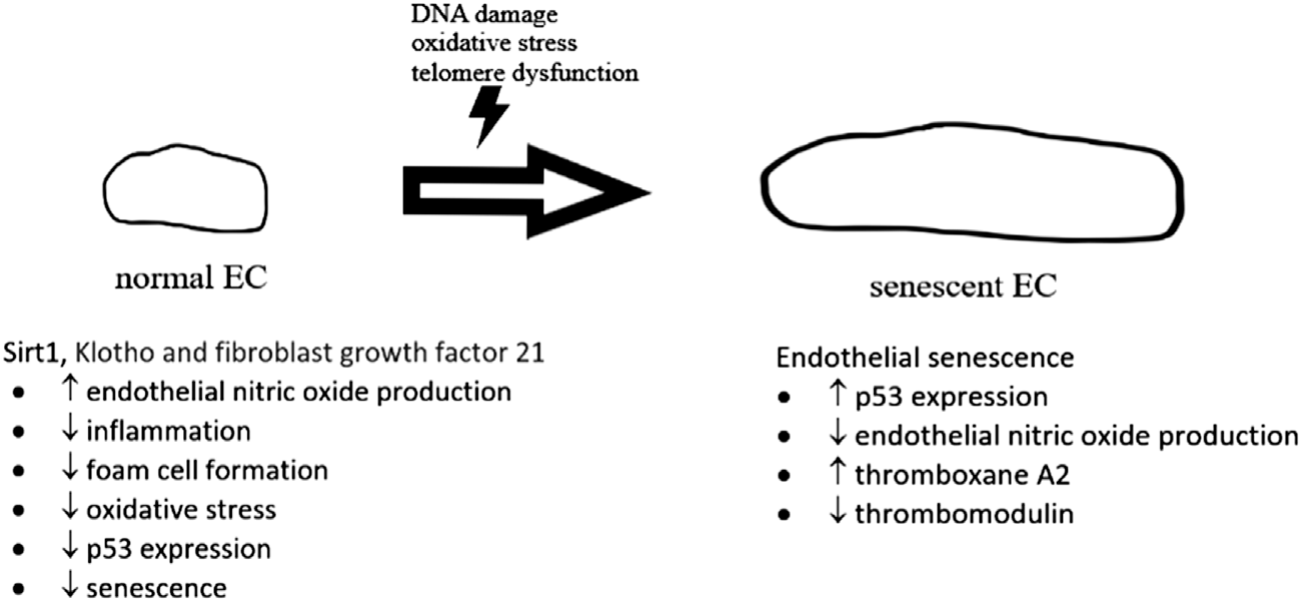
The development of normal endothelial cell (EC) to senescent EC. In normal situation, sirt 1, Klotho and fibroblast growth factor 21, etc., give protective effects on normal endothelial cells, preventing senescence. In senescent EC, expression of p53 was upregulated with a reduction of endothelial nitric oxide production.

A number of biochemical pathways involving sirtuin 1, Klotho and fibroblast growth factor 21 (Figure 2) have been found to be associated with cellular senescence (Jia et al., 2019). Cellular senescence is also regulated by p53 pathway with increased inflammatory signaling in arteries from older adults (Donato et al., 2015). p53 is found to impair endothelium-dependent vasodilatation, which is important for maintaining healthy blood flow. A study found that p53 mediates angiotensin II-induced impairment of vasodilatation (Kim et al., 2008). Another study by Kumar et al. (2011) found that *ex vivo* adenoviral overexpression of p53 of rat aortic rings impaired endothelium-dependent vasodilatation. *In vitro* studies in the same paper showed that overexpression of p53 in human umbilical vein endothelial cells suppressed Kruppel-like Factor 2 (KLF2), which was known to induce eNOS expression (Kumar et al., 2011). Diabetes was also found to induce a marked increase in endothelial p53 level as well as an impairment of endothelium-dependent vasodilatation. This impairment resulted in significantly reduction of endothelial cell-specific p53 gene deletion (Yokoyama et al., 2019). *In vitro* studies in the same paper argued that p53 inactivates eNOS through inhibiting the phosphorylation of eNOS at its Ser1177 residue, one of the important regulatory sites for eNOS activity (Yokoyama et al., 2019; Thai et al., 2021).

The roles of p53 in mediating vascular oxidative stress and pro-inflammatory responses in diabetes were studied. Exposure to high glucose promoted the expression of p53 in endothelial cells, and treatment with pifithrin-α (small molecule inhibitor of p53) or p53 siRNA attenuated oxidative stress and mRNA expression of adhesion molecules (VCAM-1, ICAM-1, E-selectin) and MCP-1 in high glucose-treated endothelial cells. Administration of pifithrin-α to diabetic mice *in vivo* significantly improved endothelium-dependent vasodilatation response of aorta (Wu et al., 2019). Interestingly, treatment with SRT2104, a sirtuin 1 activator, protects against high glucose-induced endothelial dysfunction by inhibiting p53 activity (Wu et al., 2018). In addition, SUMOylation of p53 was found to mediate disturbed flow-induced endothelial cell apoptosis (Heo et al., 2011). Disturbed flow activates PKCζ and upregulates p53 SUMOylation. SUMOylated p53 translocates from nucleus to cytoplasm, and this upregulates apoptosis (Heo et al., 2011). Subsequent studies found that increased SUMOylation of p53 promotes endothelial dysfunction and inflammation (Heo et al., 2013). The regulation and role of p53 in endothelial cell under different physiological events are summarized in Table 1.

**TABLE 1:** Summary of regulation of p53 in endothelial cell under different physiological or pathophysiological conditions.

Physiological or pathophysiological conditions	Model	Regulation of p53	Role of p53	References
Vascular function	Bovine aortic endothelial cells with laminar shear stress	Laminar shear stress → JNK ↑ → p53↑	cycle arrest ↓ and apoptosis ↓	Lin et al. (2000)
Endothelial dysfunction	Angiotensin II (Ang II) treatment in p66shcRNAi transgenic B6SJL mice transfected human umbilical vein and human aortic endothelial cells with p53	Ang II → p53 ↑	p66shc ↑ → endothelium-dependent vasodilatation ↓	Kim et al. (2008)
Endothelial dysfunction	Adenoviral overexpression of p53 in rat aortic rings overexpression of p53 human umbilical vein endothelial cells (HUVECs)	p53 ↑ → KLF2 ↓ → eNOS ↓	Endothelium-dependent vasodilatation ↓	Kumar et al. (2011)
Endothelial dysfunction	Streptozotocin (STZ)-induced diabetes in C57BL/6 mice hyperglycemia in HUVECs	High glucose (HG) → p53 ↑ → PTEN ↑ → peNOS (ser 1117) ↓	endothelium-dependent vasodilatation ↓	Yokoyama et al. (2019)
Endothelial dysfunction	High glucose treated primary endothelial cells isolated from C57BL/6 mice	HG → miR-34a ↑ → sirtuin 1 ↓ → p53 ↑	inflammation and oxidative stress ↑	Wu et al. (2019)
Endothelial dysfunction	STZ-induced diabetes in C57BL/6 mice and its isolated primary endothelial cells with SRT2104 treatment	HG → sirtuin ↓ → p53 ↑ →VCAM-1, ICAM-1,E-selectin and MCP-1 ↑	p53 deacetylation mediates SRT2104’s protection against diabetes-induced aortic endothelial dysfunction	Wu et al. (2018)
	HUVECs with disturbed flow stimulation	PKCζ ↑ → PKCζ- PIASy binding ↑ → SUMOylation of p53 ↑ → bcl-2 ↑	Apoptosis ↑	Heo et al. (2011)
Endothelial dysfunction	Primary endothelial cells isolated from SENP2 deficiency C57BL/6J mice stimulated with disturbed flow	SENP2 ↓ → SUMOylation of ERK5 and p53 ↑ → Bcl-2 ↑, eNOS ↓, KLF2 ↓, VCAM-1, ICAM-1 and E-selectin ↑	Apoptosis ↑ inflammation ↑	Heo et al. (2013)
Vascular remodeling in pulmonary hypertension	Pulmonary arterial endothelial cells isolated from mice with hypoxia-induced pulmonary hypertension (PH) and rats with monocrotaline (MCT)-induced PH	Hypoxia → HIF-2α ↑ → p53↑ → Bax/Bcl-2 ↑	Apoptosis↑ → pulmonary hypertension ↑	Wang et al. (2018)
Vascular remodeling in pulmonary hypertension	Pulmonary arterial and lung microvascular endothelial cells with DNA damage and oxidant stress	genotoxic stress (doxorubicin) → p53-PPARγ complex ↑ → PGC1A and APLN ↑ Oxidative stress → BMPR2 ↑ → p53-PPARγ complex ↑ → EPHA2 (ephrin type-A receptor 2), FHL2 (four and a half LIM domains protein 2), JAG1 (jagged 1), SULF2 (extracellular sulfatase Sulf-2), and TIGAR (TP53-inducible glycolysis and apoptosis regulator) ↑	DNA repairing, angiogenesis ↑ and apoptosis ↓ → pulmonary hypertension ↓	Hennigs et al. (2021)

## p53 and atherosclerosis

Atherosclerosis is a chronic inflammatory disorder occurring in mid-sized and large arteries. It is characterized by the excessive infiltration of lipid and inflammatory cells and resultant formation of fibrous and fatty lesions within the tunica intima (Ross, 1999; Insull, 2009; Hansson and Hermansson, 2011). The progressive growth of atherosclerotic lesions leads to the impediment of blood flow. With a rupture or erosion of an unstable plaque, the contact between the blood and materials within the plaques can quickly trigger thrombosis, leading to ischemic injury or death of the tissue to which the affected artery supplies blood. The most debilitating or fatal clinical manifestations of atherosclerosis are myocardial infarction and stroke (Insull, 2009; Hansson and Hermansson, 2011).

At the initial stages of atherosclerosis, endothelial dysfunction favors the recruitment of neutrophils and monocytes into the subendothelial space of tunica media (Bevilacqua et al., 1994; Blankenberg et al., 2003). Once recruited into the subendothelial space, monocytes mature into macrophages (Negre-Salvayre et al., 2020). The endothelium then becomes more permeable to low-density lipoprotein (LDL), leading to increased accumulation of LDL within the subendothelial space (Schwenke and Carew, 1989; von Eckardstein and Rohrer, 2009). LDL is oxidized by the reactive oxygen species (ROS) produced by the activated leukocytes within the subendothelial space, and macrophages internalize oxidized LDL (oxLDL) to become cholesterol-laden foam cells (Stocker and Keaney, 2004). oxLDL also promotes inflammation of the blood vessel (Khan et al., 1995; Sawamura et al., 1997; Miller et al., 2003). Foam cells reside within the tunica media and generate various pro-inflammatory factors including cytokines, chemokines, ROS and matrix-degrading proteases (Tabas and Bornfeldt, 2016). Moreover, pro-inflammatory cytokines can stimulate the expression inducible nitric oxide synthase (iNOS) in vascular smooth muscle cells (Chan and Fiscus, 2004) and macrophages (Depre et al., 1999). Unlike eNOS, iNOS produces a large amount of NO for a prolonged period (Cho et al., 1992). Coinciding with an increased production of ROS, iNOS-drived NO is chemically converted into other reactive nitrogen species (e.g., peroxynitrite) which is a stronger oxidant, contributing to cell damage (Martínez and Andriantsitohaina, 2009; Förstermann et al., 2017).

The number of macrophages/foam cells continues to increase and lead to growth in lesion size. In more advanced lesions, this increase in lesion size is also contributed by scavenger receptor-initiated proliferation of macrophages (Robbins et al., 2013). The overall size of lesion is determined also by the ability of macrophages to migrate away from the lesion, the rate of macrophage/foam cell death (Shi et al., 2018), and the ability of lesion macrophages to clear apoptotic or necrotic materials by efferocytosis (Tabas and Bornfeldt, 2016; Brophy et al., 2017). These processes are likely impaired in advanced lesions because macrophage/foam cell migration is impaired by increased expression of iNOS and exaggerated generation of reactive nitrogen species (Huang et al., 2014), and the efferocytosis of lesion macrophages is also known to be impaired by ROS and matrix-degrading proteases (Thorp et al., 2011). Impairment of macrophage efferocytosis allows the formation of an acellular necrotic core enriched with lipid and crystalline cholesterol released by necrotic foam cells, which is a key histopathological feature of advanced atherosclerotic lesions (Tabas and Bornfeldt, 2016). Apart from the formation of necrotic core, advanced atherosclerotic lesions also feature a fibrous cap underneath the endothelium. Upon stimulation by pro-migratory signals including growth factors (e.g., PDGF, FGF), cytokines, thrombin, extracellular matrix components or high glucose level, smooth muscle cells in tunica media acquire a synthetic phenotype, resulting in their proliferation and migration to the subendothelial space. Vascular smooth muscle cells deposit collagen causing the expansion of extracellular matrix at the subendothelial space, which appears as a fibrous cap (Gerthoffer, 2007; Brophy et al., 2017). As such, advanced lesions with necrotic core and fibrous cap are called fibro-fatty atheroma (Stary et al., 1994).

In advanced atherosclerotic plaques of human, the expression and phosphorylation of p53 was found to be elevated (Iacopetta et al., 1995; Gorgoulis et al., 2005). *In vivo* study showed that global p53 deficiency accelerated atherosclerotic plaque formation in atherosclerosis-prone apoE−/− mice (Guevara et al., 1999; Mercer et al., 2005). Subsequently, two studies showed that LDL receptor-deficient mice and APOE*3-Leiden mice transplanted with bone marrow of p53-deficient mice also had accelerated atherosclerotic plaque formation (van Vlijmen et al., 2001; Merched et al., 2003). Based on histological evidence, two of these studies found that p53 deficiency reduced cell proliferation within atherosclerotic lesions, but did not affect apoptosis there (Guevara et al., 1999; Merched et al., 2003), while one of them found that p53 deficiency reduced apoptosis but did not affect cell proliferation (van Vlijmen et al., 2001). One of these studies also demonstrated that transplant of p53-positive bone marrow to p53−/−/apoE−/− mice reduced atherosclerotic lesion formation and cell proliferation and apoptosis within atherosclerotic lesions (Mercer et al., 2005). *In vitro* experiments showed that p53 differentially regulate the proliferation and apoptosis of macrophages and smooth muscle cells. For macrophages, p53 promotes their apoptosis. However, for vascular smooth muscle cells, p53 inhibits their proliferation and limits their apoptosis *in vitro* (Mercer et al., 2005). The regulation of p53 in vascular smooth muscle cells and macrophages are summarized in Table 2 and Table 3 respectively.

**TABLE 2:** Summary of regulation of p53 in smooth muscle cell under different physiological or pathophysiological conditions.

Physiological or pathophysiological conditions	Model	Regulation of p53	Role of p53	References
Atherosclerosis	Human atheromatous arterial wall collected from patients with occlusive and aneurysmal disease	p53 ↑	Atherosclerotic plaques ↑	Iacopetta et al. (1995)
Atherosclerosis	Senescent human vascular smooth muscle cell line HVTs-SM1	p53 ↑ → ICAM-1 ↑	Atherosclerotic lesions ↑	Gorgoulis et al. (2005)
Atherosclerosis	p53 knockout in apoE−/− C57BL/6J mice	↓ p53 → smooth muscle cells and macrophages proliferation ↑	Atherosclerotic lesion ↑	Guevara et al. (1999)
Atherosclerosis	Vascular smooth muscle cell isolated from p53 knockout and ApoE knockout C57Bl6/J mice	↓ p53 → proliferation ↓ DNA damage → p53↑ → ATM/ATR substrates and P-Chk-1 ↓	Apoptosis and proliferation ↓ → aortic plaque formation ↓	Mercer et al. (2005)
Atherosclerosis	Human aortic smooth muscle cell from patients with abdominal aortic aneurysm	miR-504 → p53↓ → p21 ↓ → Bax, caspase-3, 9 and bcl-2 ↓	Proliferation and apoptosis ↓	Cao et al. (2017)
Atherosclerosis	Vascular smooth muscle cell isolated from rat/transgenic rat	Oxidative stress ↑ → p53 ↑ → p21 ↑→ IGF1R ↓	Apoptosis↑ → atherosclerotic plaques ↑	Kavurma et al. (2007)
Atherosclerosis	ApoE knockout mice with overexpressed p53	Not reported	p53↑ → plaque stability ↓ and rate of plaque rupture ↑	von der Thüsen et al. (2002)
Vascular remodeling in pulmonary hypertension	p53 global knockout mice with hypoxia stimulation	p53 ↓ → ↓ p21 and ↑ HIF-1α	Vascular remodeling ↑ → pulmonary hypertension ↑	Mizuno et al. (2011)
Vascular remodeling in pulmonary hypertension	p53 or p21 knockout C57Bl/6j with Nutlin-3a treatment and exposed to chronic hypoxia	Hypoxia + nutlin-3a → MDM2 → p53 ↑ → p21 ↑ → Bcl2, Bax ↑ and PUMA ↓ (in lung)	apoptosis ↓ and proliferation ↓ → pulmonary hypertension ↓	Mouraret et al. (2013)
Vascular remodeling in pulmonary hypertension	Pulmonary artery smooth muscle cells isolated from p53 condition knockout C57BL/6NCr	No effect	No effect	Wakasugi et al. (2019)
Vascular remodeling in pulmonary hypertension	Pulmonary artery (PA)-smooth muscle cells isolated from MCT-induced pulmonary hypertension rats	Baicalein and p53 → Bax, caspase-3 and bcl-2 ↑	Apoptosis ↑ → pulmonary artery remodeling ↓	Teng et al. (2022)
Vascular remodeling in pulmonary hypertension	Pulmonary arterial smooth muscle cells isolated from mice with PH and rats with MCT-induced PH	Hypoxia → HIF-1α → p53 ↓ → transient receptor potential channels 1 and 6 ↓	Proliferation ↑ → pulmonary hypertension ↑	Wang et al. (2018)

**TABLE 3:** Summary of regulation of p53 in macrophage during atherosclerosis.

Physiological effect	Model	Regulation of p53	Role of p53	References
Atherosclerosis	Macrophages isolated from p53 knockout and ApoE knockout C57Bl6/J mice	Not reported	p53 ↑ → apoptosis ↑	Mercer et al. (2005)
Atherosclerosis	APOE*3-Leiden transgenic C57BL/kh mice with p53 knockout	Not reported	p53 ↓ → apoptosis ↓ → atherosclerotic lesion area ↑	van Vlijmen et al. (2001)
Atherosclerosis	LDL receptor- C57BL/6J deficient mice	Not reported	p53 ↓ in macrophage proliferation ↑ → Atherosclerotic lesion area ↑	Merched et al. (2003)

A more recent study showed that apoE−/− mice with smooth muscle-specific p53 gene knockout had no significant difference in overall size of atherosclerotic lesions, compared to apoE−/− mice with p53 in their smooth muscle. Interestingly, this study also found that apoE−/− mice with smooth muscle-specific p53 deficiency had a significantly higher number of smooth muscle cells within the fibrous caps of atherosclerotic plaques, and the *in vitro* experiments of the same study showed that p53 deficiency enhanced vascular smooth muscle cell migration and invasion (Cao et al., 2017). An *in vitro* study showed that p53 negatively regulates the expression of IGF1 receptor, which is a key mechanism for vascular smooth muscle cell survival and proliferation (Kavurma et al., 2007). IGF1 receptor activation is also important for smooth muscle cell migration (Beneit et al., 2016). Therefore, it is plausible that p53 limited vascular smooth muscle cell migration through downregulating IGF1 receptor expression. One study looked at the effect of p53 on the stability of advanced atherosclerotic lesion through collar-induced carotid atherogenesis in apoE−/− mice, overexpression of p53 reduced plaque stability and promoted the rate of plaque rupture were observed (von der Thüsen et al., 2002). Depending on the culprit vessel, plaque rupture can lead to myocardial ischemia or cerebral ischemia. For instant, cerebral vasospasm secondary to cerebral aneurysm and hemorrhage stroke can lead to hypoperfusion resulting in delayed cerebral ischemic deficits (Sun and Feinberg, 2021), cerebral-vasospasm]. An *in vivo* study suggested that p53 plays an important role in the etiology of vasospasm which contributed directly to the alteration of the blood brain barrier (BBB) integrity and cerebral oedema development during the first 72 h of subarachnoid hemorrhage (SAH) (Cahill et al., 2006). Furthermore, recent study showed that angiopoietin-1 (Ang-1) inhibited the p53-mediated endoplasmic reticulum stress and apoptosis in the vascular endothelial cells in rats with SAH (Wei et al., 2022). These studies highlighted the roles of p53 in cerebral vasculature and its potential as a treatment target for SAH.

## p53 and vascular remodeling in pulmonary hypertension

Pulmonary hypertension is a disease with high morbidity and mortality. After the sixth World Symposium on Pulmonary Hypertension in 2018, the diagnostic criteria of pulmonary hypertension was defined as mean pulmonary arterial pressure (mPAP) > 20 mmHg and pulmonary vascular resistance ≥3 Wood Units (Simonneau et al., 2019). Pulmonary hypertension is primarily associated with hemodynamic alterations resulting from remodeling of pulmonary arteries and veins, such as increased media/lumen ratio or increased media cross-sectional area. Pulmonary arterial smooth muscle cells and endothelial cells are the major players involved in vascular remodeling. Vascular remodeling is driven by four cellular processes: cell proliferation, cell apoptosis, cell migration, and synthesis or degradation of extracellular matrix that can be triggered by various growth factors, vasoactive substances and stimuli such as chronic hypoxia and DNA damage (Tuder, 2017).

Pulmonary arterial smooth muscle cells and endothelial cells are the major players involved in vascular remodeling. Vascular remodeling is driven by four cellular processes: cell proliferation, cell apoptosis, cell migration, and synthesis or degradation of extracellular matrix that can be triggered by various growth factors, vasoactive substances and stimuli such as chronic hypoxia and DNA damage (Tuder, 2017).

In the pathogenesis of pulmonary hypertension, conditions such as chronic hypoxia, activation of voltage-gated calcium channels, and extracellular calcium-sensing receptors contributed to vascular remodeling by promoting pulmonary arterial smooth muscle cell proliferation and retarding apoptosis of endothelial cells (Smith et al., 2016; Xiao et al., 2017; Shimoda, 2020). p53, a master regulator of cell cycle arrest, apoptosis, cell proliferation and DNA repair etc., was found to crosstalk with hypoxia-inducible transcription factors. Therefore, it is of interest to investigate whether p53 plays a role in cellular processes that drive vascular remodeling, particularly for pulmonary arterial smooth muscle cells and endothelial cells.

With the murine model of chronic hypoxia-induced pulmonary hypertension, it was found that pulmonary hypertension was promoted in p53 global knockout mice, with increased vascular remodeling, upregulated hypoxia-inducible factor 1 alpha (HIF-1α) expression and downregulated p21 expression in the pulmonary arterial smooth muscle cell (PASMC) (Mizuno et al., 2011). Mouraret et al. (2013) demonstrated the administration of Nutlin-3a to chronically hypoxic mice markedly increased p53 expression in lungs and partially reversed pulmonary hypertension and attenuated vascular remodeling. The partial reversal of pulmonary hypertension was not apparent in chronically hypoxic p53 global knockout mice and p21 global knockout mice, suggesting that p53 an p21 are required in Nutlin-3a-induced partial reversal of pulmonary hypertension (Mouraret et al., 2013). PASMC-specific gain or loss of p53 function does not affect hypoxia-induced pulmonary hypertension in mice, suggesting that the modulation of p53 signaling in other cells is required to bring about phenotypic change in the murine hypoxia-induced pulmonary hypertension (Wakasugi et al., 2019). A recent study by Teng et al. (2022) found that PASMC-targeted co-delivery of baicalein and p53 reversed pulmonary artery remodeling in monocrotaline-treated rats and promoted PASMC apoptosis *via* Bax/BCl2/Caspase 3 signaling pathway. However, PASMC-targeted delivery of p53 alone has no significant impact on vascular remodeling.

Recent studies provided experimental evidence delineating the complex functional role and regulation of p53 in pulmonary vessels exposed to hypoxia. Wang et al. (2018) observed that p53 expression is decreased in PASMC under hypoxia, while the opposite occurs for pulmonary arterial endothelial cells (PAEC). The proposed mechanism for this divergent regulation of p53 is that hypoxia increases the expression of HIF-1α in PASMC, while it increases the expression of hypoxia-inducible factor 2 alpha (HIF-2α) instead for PAEC. These two hypoxia-inducible factors affect p53 expression in opposite manners. While an increased expression of HIF-1α in PASMC downregulates p53 expression, an increased HIF-2α in PAEC instead upregulates p53 expression (Wang et al., 2018). Functionally, hypoxia-induced downregulation of p53 in PASMC enhanced PASMC proliferation by inactivating transient receptor potential channels 1 and 6; while the upregulation of p53 in PAEC under hypoxia promoted PAEC apoptosis. Interestingly, a more recent study found that upregulation of p53 together with Peroxisome Proliferator-Activated Receptor-gamma (PPAR-γ) in pulmonary arterial and microvascular endothelial cells can promote angiogenesis and regeneration of pulmonary microvessels (Hennigs et al., 2021).

## Conclusion

Abundant evidence supports the involvement of p53 in regulating vascular function and pathogenesis of cardiovascular diseases, suggesting that p53 is of great potential as a drug target for cardiovascular diseases. However, *in vivo* studies with global or cell type-specific targeting of p53 produced differential effects on the pathogenesis of atherosclerosis and pulmonary hypertension in experimental models. Further *in vitro* studies also demonstrated diverse functional roles and regulatory mechanism of p53 in different vascular cells and macrophages. Therefore, further studies on cell-type specific function and regulation of p53 in various disease models are warranted, so that optimal p53-targetting therapy can be designed.
